# Exposure–Response Analysis for Aripiprazole Once‐Monthly in Patients Diagnosed With Bipolar I Disorder

**DOI:** 10.1002/cpdd.1580

**Published:** 2025-08-22

**Authors:** Xiaofeng Wang, Luann Phillips, Matthew Harlin, Karimah S. Bell Lynum, Frank Larsen, Pedro Such, Jessica Madera‐McDonough, Murat Yildirim, Ric M. Procyshyn, Craig Chepke, Julie Passarell

**Affiliations:** ^1^ Pharmacometrics Otsuka Pharmaceutical Development & Commercialization, Inc. Princeton NJ USA; ^2^ Simulations Plus, Inc. Clinical Pharmacology and Pharmacometrics Business Unit Buffalo NY USA; ^3^ Discovery Research, Early Phase and Translational Medicine Otsuka Pharmaceutical Development & Commercialization, Inc. Princeton NJ USA; ^4^ Global Medical Affairs Otsuka Pharmaceutical Development & Commercialization, Inc. Princeton NJ USA; ^5^ Clinical & Quantitative Pharmacology H. Lundbeck A/S Valby Denmark; ^6^ Global Medical Affairs H. Lundbeck A/S Valby Denmark; ^7^ Department of Psychiatry University of British Columbia British Columbia Mental Health and Substance Use Research Institute Vancouver BC Canada; ^8^ Excel Psychiatric Associates, P.A. Huntersville NC USA

**Keywords:** antipsychotic treatment, long‐acting injectable, pharmacokinetic modeling, population pharmacokinetics

## Abstract

Aripiprazole once‐monthly (AOM) is approved for the maintenance monotherapy treatment of bipolar I disorder (BP‐I) in adults. A previously developed population pharmacokinetic model was validated by applying it to data from patients diagnosed with BP‐I. The model was then used to generate aripiprazole exposures for an exposure‐response model intended to describe the relationship between aripiprazole exposure and time to recurrence of any mood episode in patients with BP‐I. The BP‐I data were best described by a continuous model in which higher aripiprazole exposure was associated with a lower risk of recurrence. For each 1 ng/mL increase in aripiprazole plasma concentration, the predicted hazard for recurrence decreased by 0.34%. An aripiprazole plasma concentration of 95 ng/mL (clinically relevant in schizophrenia) was tested in a separate categorical model and was a significant predictor of time to recurrence (*P *= .0270), with a 36% (1.55‐fold) decrease in the predicted risk of recurrence with an aripiprazole concentration of ≥95 versus <95 ng/mL. Plasma aripiprazole concentration was identified as an important predictor of recurrence in patients diagnosed with BP‐I treated with AOM, highlighting the importance of maintenance therapy. A 95 ng/mL threshold is relevant in BP‐I, but with a smaller effect size compared with that in schizophrenia.

Aripiprazole once‐monthly (AOM) is a long‐acting injectable (LAI) antipsychotic that has received regulatory approval for the treatment of schizophrenia and the maintenance monotherapy treatment of bipolar I disorder (BP‐I) in adults in many regions, including the United States and Canada. AOM contains aripiprazole in its monohydrate polymorphic form (7‐[4‐[4‐(2,3‐dichlorophenyl)‐1‐piperazinyl] butoxy]‐3,4 dihydrocarbostyril monohydrate).^1^ AOM exhibits slow and prolonged absorption, with steady‐state concentrations achieved after 4 monthly doses.^1^ Aripiprazole is metabolized mainly by the liver via 3 principal biotransformation pathways: dehydrogenation, hydroxylation (both mediated by cytochrome P450 [CYP]3A4 and CYP2D6), and CYP3A4‐mediated N‐dealkylation.^1^ Excretion of aripiprazole occurs primarily via the liver and to a lesser extent the kidneys.^1^ Medications that induce or inhibit CYP3A4 and CYP2D6 can affect plasma levels of aripiprazole owing to the effect of these enzymes on the drug's metabolism.^1^

In a prior exposure–response (E‐R) time‐to‐relapse model for AOM specific to schizophrenia, a clear relationship between aripiprazole plasma concentrations of 95 ng/mL and the risk of relapse was demonstrated. Specifically, a patient with a diagnosis of schizophrenia and a predicted aripiprazole minimum concentration (C_min_) of ≥95 ng/mL was 4.41 times less likely to relapse than a patient with a C_min_ of <95 ng/mL.^2^ Since the publication of these data, a 95 ng/mL threshold has been recognized as a minimum therapeutic concentration and clinically relevant target,^3^ serving as a relevant benchmark for evaluating aripiprazole concentration sufficiency with different LAI formulations of aripiprazole.^3^ This includes a newer LAI formulation of aripiprazole—aripiprazole 2‐month ready‐to‐use 960 mg (Ari 2MRTU 960)—which is associated with a plasma aripiprazole concentration that exceeded 95 ng/mL across the full 2‐month dosing interval in both a clinical trial setting and in population pharmacokinetic (popPK) modeling and simulation analyses.^4^, ^5^, ^6^

While the results of the prior E‐R analysis have played an important role in defining sufficient aripiprazole exposure in schizophrenia, it remains unclear whether a 95 ng/mL efficacy threshold also applies in patients diagnosed with BP‐I. Even though AOM is indicated for the treatment of both diseases—and the central goal in both settings is relapse prevention—schizophrenia and BP‐I are distinct mental health conditions. With this in mind, the current analysis aimed to quantify the E‐R relationship between AOM 400 mg (AOM 400) and time to recurrence of any mood episode in patients diagnosed with BP‐I.

## Materials and Methods

The current analysis was conducted in 2 parts. First, a previously developed popPK model^2^ was externally validated using plasma aripiprazole concentration data (collected in a clinical trial of AOM 400 in patients diagnosed with BP‐I) to generate exposures for use in E‐R modeling. Second, an E‐R model was developed to describe the relationship between aripiprazole exposure and time to recurrence of any mood episode in patients diagnosed with BP‐I.

Pharmacokinetic and efficacy data for the current analysis were derived from a study that evaluated the efficacy, safety, and tolerability of AOM 400 (Abilify Maintena) as maintenance treatment for BP‐I (NCT01567527). Full details of the trial design and eligibility criteria have been reported previously^7^ and are summarized in Appendix S1. In brief, the trial was conducted in 4 phases: oral aripiprazole conversion (if required [Phase A, 4–6 weeks]), oral aripiprazole stabilization (Phase B, 2–8 weeks), single‐blind AOM stabilization (Phase C, 12–28 weeks), and a double‐blind, withdrawal phase in which patients were randomized to either continue maintenance AOM 400 or were switched to placebo (Phase D, 52 weeks).^7^ The study was conducted in multiple sites worldwide, and the protocol was approved by central or local institutional review boards, as detailed in Appendix S1. Written informed consent was obtained from all patients (or their guardians or legal representatives, where applicable).^7^ The trial was conducted in compliance with the International Conference on Harmonization Guideline for Good Clinical Practice^7^ and in accordance with the principles expressed in the Declaration of Helsinki. The study drug was administered by site personnel.^7^

### Pharmacokinetic Data and Analytic Assay Methods

Plasma samples for the measurement of aripiprazole concentrations were collected prior to oral aripiprazole or AOM dosing on weeks 1, 2, 4, 12, and 28 of Phase C and prior to AOM dosing on weeks 4 and 8 of Phase D of the trial.

Aripiprazole (OPC‐14597), plus the internal standard (OPC‐14597‐d8), were extracted from human plasma using liquid–liquid extraction and analyzed using high‐performance liquid chromatography (HPLC) with tandem mass spectrometry. The HPLC system comprised an LC‐20AD (Shimadzu, Kyoto, Japan) equipped with a Betasil Silica‐100 analytical column (50 × 3 mm, 5 µm particle size; Thermo Fisher, Massachusetts, USA) maintained at 35°C. The mobile phase consisted of 10 mM ammonium acetate (mobile phase A) and acetic acid:acetonitrile (1:1000, v:v) (mobile phase B), delivered at a flow rate of 1 mL/min. Detection was performed using an API 5000 triple quadrupole mass spectrometer (Sciex, Massachusetts, USA) with positive ion electrospray ionization. Quantitation was conducted in multiple reaction monitoring mode, with response measured as peak area ratios (aripiprazole/internal standard). Data processing was performed using Analyst software (Sciex, Massachusetts, USA). Samples were quantitated against calibration standards prepared in plasma and processed like the samples. In addition, quality control samples were processed to monitor analytical performance. Quantitation was performed using a linear regression model with 1/x^2^ weighting over a range of 0.500 (lower limit of quantitation [LLOQ]) to 500 ng/mL for aripiprazole. The within‐day and between‐day precision and accuracy for the LLOQ, and the low, mid, and high quality control (QC) samples met the prespecified acceptance criteria of ≤15% relative standard deviation (SD) and between 85.0% and 115% of nominal concentration for QCs, and ≤20% and 80.0% and 120.0% of nominal for the LLOQ, respectively. The dates, times, and amounts of doses, starting with the last dose of Phase B through to the end of the trial, were combined with the dates, times, and plasma concentrations to create the popPK dataset. Missing dose times for oral aripiprazole were imputed based on the time of the prior dose that was recorded for the patient. Plasma concentrations following an AOM dose with a missing time were excluded from the analysis. Plasma concentrations that were below the lower limit of quantitation (BLQ) were also excluded from the popPK modeling (consistent with the exclusion of BLQ samples when the popPK model was initially developed).

Data for baseline body mass index (BMI), sex, CYP2D6 metabolizer status (ultra, extensive, and intermediate metabolizers [grouped] vs. poor metabolizers vs. missing), time‐varying presence or absence of concomitant use of quinidine or strong CYP2D6 inhibitors, and presence or absence concomitant use of ketoconazole or strong CYP3A4 inhibitors were also included in the popPK analysis dataset.

### Efficacy Data

The efficacy endpoint of the trial was time from randomization to recurrence of any mood episode during Phase D of the trial, defined as meeting any of the following criteria at any time: (1) hospitalization for any mood episode; (2) Young Mania Rating Scale (YMRS) total score ≥15, Montgomery–Åsberg Depression Rating Scale (MADRS) total score ≥15, and/or Clinical Global Impression‐Bipolar Version‐Severity score >4 (overall score); (3) serious adverse event (AE) of worsening disease (ie, BP‐I); (4) discontinuation due to lack of efficacy or discontinuation due to an AE of worsening disease; (5) clinical worsening with the need for the addition of a mood stabilizer, antidepressant treatment, antipsychotic medication, and/or an increase greater than the allowed benzodiazepine doses for treatment of symptoms of an underlying mood disorder; or (6) active suicidality, defined as a score of ≥4 on MADRS item 10 or an answer of “yes” for questions 4 or 5 of the Columbia‐Suicide Severity Rating Scale.

### Model Development Process: Population Pharmacokinetic Modeling

As a first step, external validation of a previously developed 3‐compartment popPK model for oral aripiprazole and AOM was undertaken by applying it to data from the BP‐I trial described above, with the model then used to calculate exposures for use in the E‐R model. All popPK modeling was performed using nonlinear mixed effects modeling implemented by the computer program NONMEM, Version 7, Level 3.0 (ICON Development Solutions; Hanover, MD, USA).

Details of the previously developed popPK model have been published elsewhere^2^ and are outlined in Appendix S2. The prior model was developed using data from individuals in the active treatment groups of 5 clinical trials of oral aripiprazole or AOM (6153 aripiprazole concentrations from 663 subjects [healthy volunteers, n  =  52; patients diagnosed with schizophrenia or schizoaffective disorder, n = 611]), with external validation using data from a sixth trial of AOM (896 aripiprazole concentrations from 251 patients with schizophrenia). Because the distributions of the patient descriptors that were statistically significant predictors of aripiprazole popPK model parameters (ie, sex, BMI, and CYP2D6 metabolizer status) for patients with schizophrenia or schizoaffective disorder were expected to be similar to those for patients diagnosed with BP‐I, the previous popPK model was anticipated to also fit the data from the current BP‐I trial.

Validation of the previously developed popPK model included 3 steps: (1) application of the previously developed popPK model to the BP‐I trial data; (2) quantitative predictive performance validation; and (3) visual predictive check validation. Full details of each step are described in Appendix S3. Following validation, the model was used to calculate model‐predicted exposures, which were used in the development of the E‐R model (see below).

### Model Development Process: Development of the Exposure–Response Model

Next, a survival analysis was used to describe the E‐R relationship between aripiprazole exposure and time to recurrence of any mood episode. The general procedure for the development of the E‐R model included 4 stages: (1) exploratory graphical analysis; (2) base structural model development incorporating model‐predicted drug exposure; (3) evaluation of covariate effects of age (years), BMI (kg/m^2^), baseline MADRS total score, baseline YMRS total score, sex, and race; and (4) model evaluation. Full details of each step are described in Appendix S4. E‐R modeling was performed using the survival package implemented using R Version 4.1.3 (R Foundation for Statistical Computing; Vienna, Austria).

The dataset used for the E‐R analyses included information from the double‐blind, placebo‐controlled phase of the BP‐I trial (Phase D). Time to recurrence of any mood episode, treatment assignment (placebo, AOM 300, or AOM 400), age, baseline BMI, race, sex, baseline YMRS total score, baseline MADRS total score, and popPK model‐predicted pharmacokinetic exposures were combined into the E‐R analysis dataset. The selected exposure metric was the popPK model‐predicted aripiprazole plasma concentration 672 hours (28 days) after the first dose of AOM (C_tau_) in Phase D of the study, representing the steady‐state value for the aripiprazole arm and the rate of the initial washout for the placebo arm. Since the washout of AOM is longer than 672 hours, C_tau_ was also predicted for patients randomized to placebo during Phase D to capture the plasma concentration of drug remaining from Phase C doses. Model‐predicted C_tau_ during Phase D of the trial was calculated using numerical integration, using the individual Bayesian popPK model parameter estimates for each patient and the differential equations describing the popPK model. For patients who never experienced a recurrence of mood episode during the trial or exited the trial for any other reason (censored), the time was set to the number of days the patient was observed during Phase D of the trial.

## Results

### Validation of the Population Pharmacokinetic Model

The analysis dataset for the popPK model comprised 1907 aripiprazole plasma concentrations obtained from 420 patients enrolled in Phases C and D of the BP‐I trial. Baseline demographic data for the popPK population are shown in Appendix S3. (The analysis dataset excluded 50 samples collected prior to oral aripiprazole steady‐state conditions at the end of Phase B [steady‐state concentrations were a requirement for model initialization].) Predictive performance and prediction‐corrected visual predictive check (pcVPC) evaluations using data from patients diagnosed with BP‐I indicated that the previous popPK model accurately predicted plasma aripiprazole concentrations following administration of AOM during the double‐blind phase of the BP‐I trial (minimum of 2 doses of AOM) with better than required accuracy and bias. Full results are described in Appendix S3.

### Exposure–Response Model

Overall, 265 patients from Phase D of the trial were included in the E‐R dataset; of these, 132 patients were randomized to AOM and 133 patients were randomized to placebo. Baseline demographic data for patients included in the E‐R dataset are shown in Appendix S4. All characteristics of the current BP‐I population were similar to those of the previous analysis population (ie, patients with schizophrenia), except for a greater proportion of female patients (∼60% vs. 38%). The mean (SD) number of samples per patient was 4.7 (1.7).

**Figure 1:**
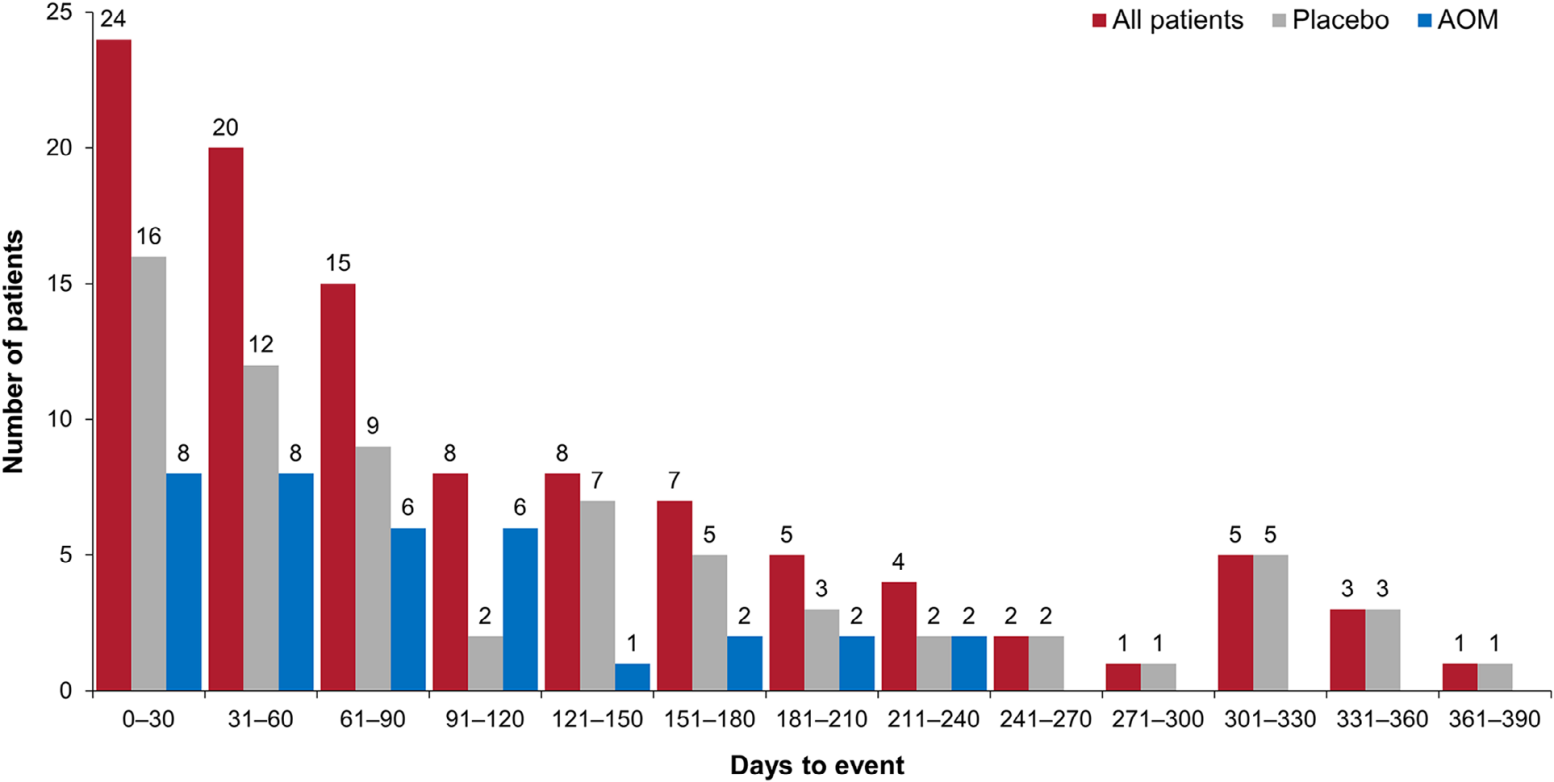
Distributions of days to recurrence of any mood episode, overall and by treatment. The number of patients is shown above each bar. AOM, aripiprazole once‐monthly.

**Figure 2:**
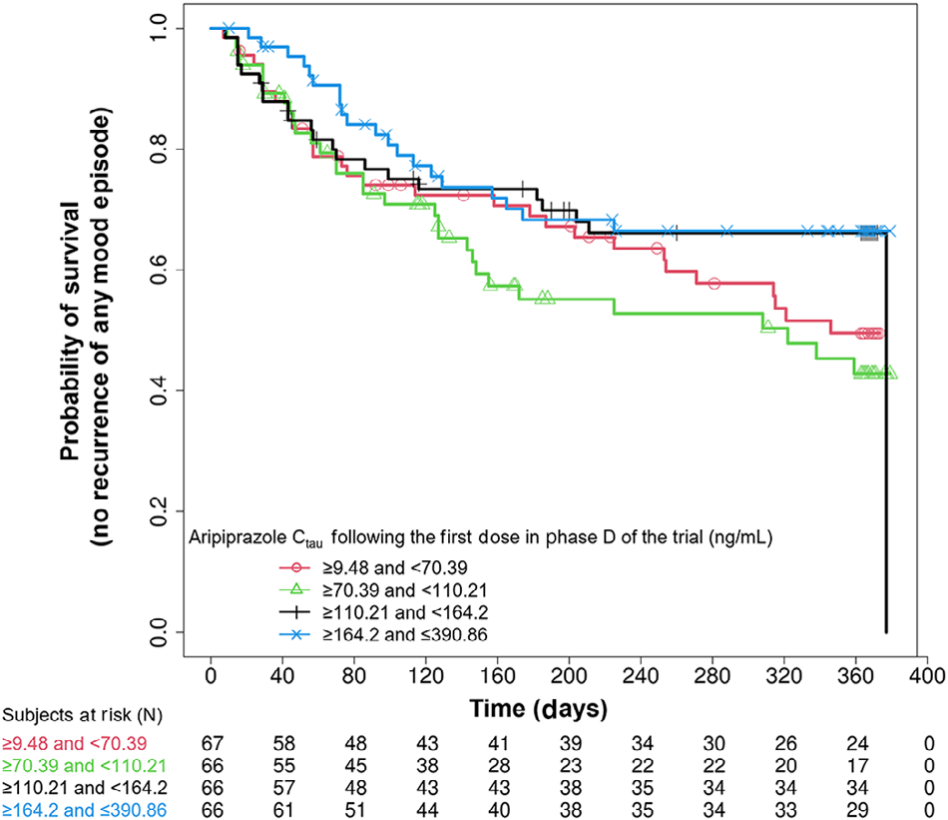
Kaplan–Meier plots of recurrence of any mood episode versus time, by quartiles of aripiprazole exposure. C_tau_, aripiprazole concentration 672 hours post dose; N, number of patients.

Exploratory data analysis showed that the mean (SD) C_tau_ following the first dose in Phase D was 170.5 (71.6) ng/mL for patients in the AOM treatment group and 78.7 (36.1) ng/mL for patients in the placebo group (this represents the washout from the last AOM dose in Phase C [approximately 1344 hours (56 days) post the last active treatment dose]). Data regarding the number of patients with a recurrence of any mood episode and the associated C_tau_, stratified by having the event and censored are shown in Table 1. There was a larger percentage of placebo recipients (51.1%) versus AOM recipients (26.5%) with an event. In the AOM treatment group, mean C_tau_ was higher for censored patients versus patients with an event. Conversely, in the placebo group, mean C_tau_ was higher for the event group; however, the SD of C_tau_ was larger than the mean C_tau_, indicating a large amount of variability. As shown in Table 1, 48.9% and 73.5% of placebo and aripiprazole recipients, respectively, were censored (ie, no mood episode). The number of days to the recurrence of any mood disorder, stratified by treatment and for all patients with an event is displayed in Figure 1. Approximately 65% of the events occurred within approximately the first 120 days of treatment during Phase D. Kaplan–Meier (KM) plots of the probability of recurrence of any mood episode versus time, by quartiles of aripiprazole exposures, are presented in Figure 2. As shown in this plot, there was an E‐R relationship indicated by a lower probability of recurrence of any mood episode for the 2 highest versus the 2 lowest quartiles of exposure.

**Table 1:** Summary Statistics of the Occurrence of Any Mood Episode and Associated C_tau_ Statistics

	Treatment group	Censor^a^ (n = 162)	Event (n = 103)	Overall (n = 265)
Treatment, n (%)	AOM	97 (73.5)	35 (26.5)	132 (100.0)
	Placebo	65 (48.9)	68 (51.1)	133 (100.0)
Mean (SD) aripiprazole C_tau_ following the first dose in Phase D of the trial, ng/mL	AOM	187.2 (81.3)	163.3 (55.9)	170.5 (71.6)
	Placebo	16.4 (42.6)	52.6 (58.6)	78.7 (36.1)

AOM, aripiprazole once‐monthly; C_tau_, aripiprazole concentration 672 hours post dose; n, number of patients; SD, standard deviation.

a Patients who never experienced a recurrence of mood episode during the trial or who exited the trial for any other reason.

The correlation between time to recurrence of any mood episode and the Schoenfeld residuals was tested and was found not to be significant (*P* = .8173), indicating that a semi‐parametric Cox proportional hazards model was the appropriate functional form for a base structural model incorporating aripiprazole C_tau_. Separate univariate models were used to evaluate aripiprazole C_tau_ as a linear and as a log‐linear predictor of the probability of time to recurrence of any mood episode. The linear function of C_tau_ was the most statistically significant (*P* = .0170) predictor and was accepted into the E‐R model. None of the tested covariates were statistically significant predictors of time to recurrence of any mood episode in the presence of aripiprazole C_tau_; as such, no covariate effects were included in the E‐R model.

The final E‐R model for time to recurrence of any mood episode was a semi‐parametric Cox proportional hazards model including the effect of aripiprazole C_tau_ following the first dose in Phase D, per the equation in Appendix S4. A higher aripiprazole C_tau_ following the first dose in Phase D of the trial was associated with a higher probability of survival (ie, a lower risk of recurrence of any mood episode). The parameter estimates and corresponding precision of the estimates for the final E‐R model of time to recurrence of any mood episode were as follows: the coefficient for the effect of C_tau_ was −0.00346 (relative standard error, 44%), the hazard ratio (HR) was 0.99655 with a 95% confidence interval (CI) of 0.9936‐0.9995, and the *P* value was .0230. The model indicated that for each 1 ng/mL increase in aripiprazole C_tau_ following the first dose in Phase D of the trial, the predicted hazard for recurrence of any mood episode decreased by 0.34%.

An aripiprazole C_tau_ of 95 ng/mL was tested as a new model (categorical) in the BP‐I population and was found to be a significant predictor of time to recurrence of any mood episode (*P*‐value = .0270). The HR for the effect of the aripiprazole cut‐off on the time to recurrence of any mood episode was 0.6450 (95% CI, 0.4372–0.9514), indicating that when the aripiprazole C_tau_ following the first dose in Phase D of the trial was ≥95 ng/mL versus <95 ng/mL, the predicted risk of recurrence of any mood episode decreased by 36%, or 1.55‐fold (1/0.6450).

**Figure 3:**
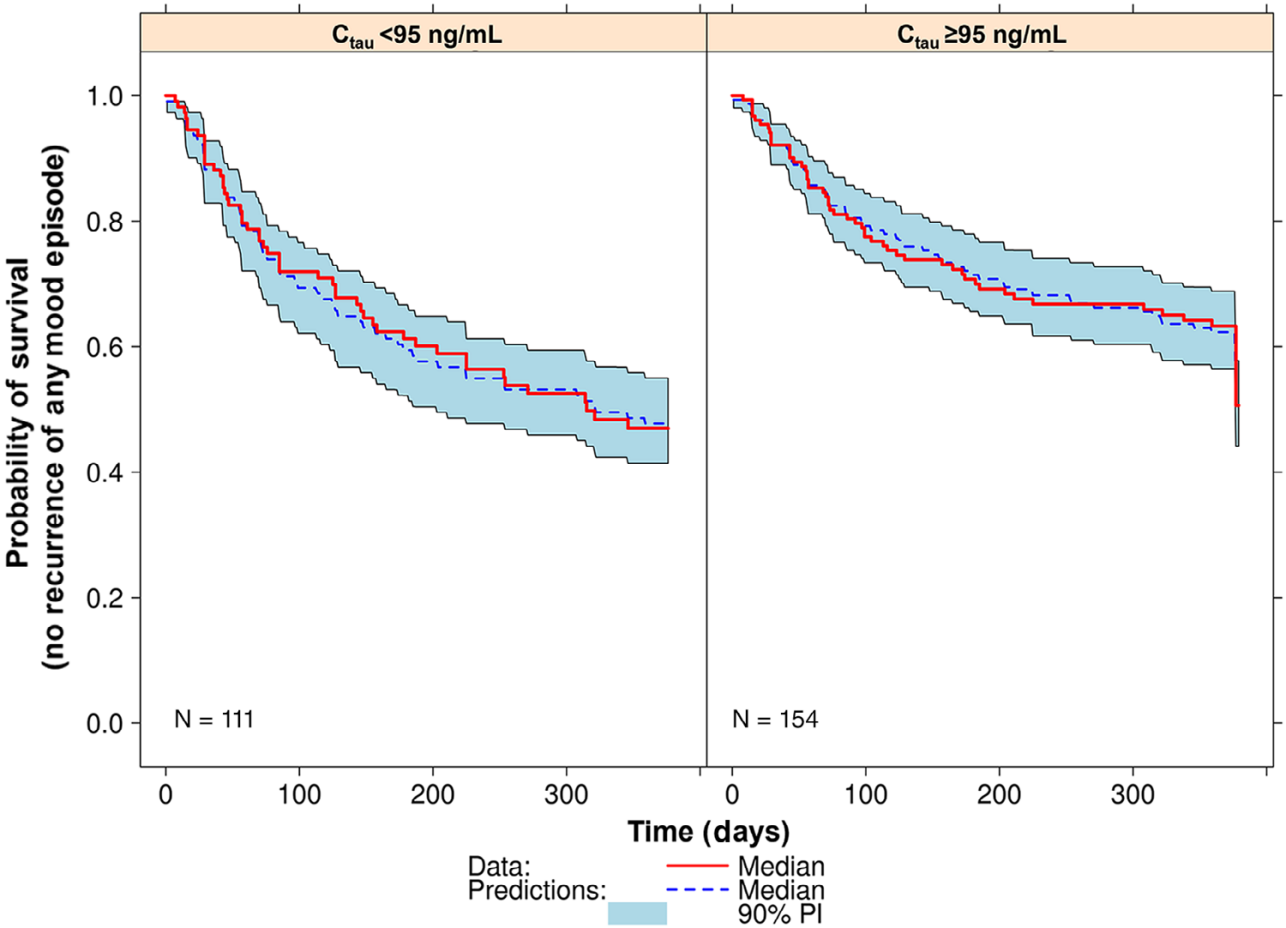
Visual predictive check plots of the simulated probability of survival versus days with Kaplan–Meier estimates of the observed data overlaid, stratified by aripiprazole exposure groups (<95 ng/mL and ≥95 ng/mL). C_tau_, aripiprazole concentration 672 hours post dose; N, number of patients; PI, prediction interval.

A visual predictive check of the final continuous model (not the cut‐off model) was performed and is illustrated in Figure 3, stratified by an aripiprazole C_tau_ of 95 ng/mL following the first dose in Phase D of the trial. The plots illustrated a good concordance between the model‐based simulations and the observed data‐based estimates of events by the C_tau_ group. The KM curves (observed data) were within the 90% prediction intervals and tracked very well with the median probability of no recurrence of mood episode across time, indicating that that the model was reliable and accurate for predicting the likelihood of recurrence of any mood episode based on drug concentration in patients diagnosed with BP‐I.

## Discussion

The overarching goal of the current analysis was to determine the relationship between predicted aripiprazole exposure and time to recurrence of any mood episode in patients diagnosed with BP‐I and to ascertain whether the aripiprazole concentration threshold of 95 ng/mL demonstrated for schizophrenia is also applicable for BP‐I. Per the E‐R analysis, the BP‐I data were best described by a model in which higher aripiprazole exposures treated continuously were associated with a lower risk of recurrence of any mood episode. A separate categorical model demonstrated that 95 ng/mL was a significant predictor of time to recurrence of any mood episode in the BP‐I population, although the effect size was smaller compared with that in schizophrenia. A summary of these findings and relevant considerations for clinicians is shown in Table 2, with additional context provided below.

**Table 2:** Considerations for Clinicians Treating Patients Diagnosed With BP‐I With AOM

What is the E‐R relationship for AOM in patients diagnosed with BP‐I?	The E‐R relationship was best described by a continuous model, with higher aripiprazole exposures associated with a lower risk of recurrence of any mood episode.
Is a 95 ng/mL concentration threshold relevant in patients diagnosed with BP‐I treated with AOM?	In a separate (categorical) model, an aripiprazole plasma concentration of 95 ng/mL was a significant predictor of time to recurrence of any mood episode. The effect size of a 95 ng/mL cut‐off in BP‐I was smaller compared with that in schizophrenia. The reasons for this are unclear but could reflect differences in disease symptomatology.
Is any other concentration threshold relevant in patients diagnosed with BP‐I treated with AOM?	Concentration thresholds other than 95 ng/mL were evaluated but none described the data better than a continuous model.
What does the E‐R data mean in terms of therapeutic drug monitoring?	The E‐R data do not indicate a general need for therapeutic drug monitoring. This is consistent with the label for AOM and clinical trial data indicating sufficiently high plasma aripiprazole concentrations are delivered in patients with BP‐I treated with AOM.^a^ ^,^ ^5^ Monitoring on a case‐by‐case basis in patients with an inadequate clinical response to AOM remains relevant. In the event of subtherapeutic exposure to aripiprazole (ie, <95 ng/mL), suitable management strategies should be implemented (eg, adherence support interventions, oral aripiprazole supplementation, addition of a CYP2D6 inhibitor [if indicated]).
What does the E‐R data mean in terms of treatment adherence?	The E‐R data demonstrate plasma aripiprazole concentrations as a predictor of mood recurrence in patients diagnosed with BP‐I. Proactive adherence monitoring and support are encouraged to ensure that patients maintain consistent levels of plasma aripiprazole.

AOM, aripiprazole once‐monthly; BP‐I, bipolar I disorder; CYP, cytochrome P450; E‐R, exposure–response.

a Data refer to patients with BP‐I or schizophrenia.

In the first step of the current analysis, a previously developed popPK model for oral aripiprazole and AOM, built using data from aripiprazole concentration records from healthy volunteers and patients with schizophrenia or schizoaffective disorder,^2^ was externally validated by applying it to plasma aripiprazole concentration data collected during a clinical trial conducted in patients diagnosed with BP‐I.^7^ Predictive performance and pcVPC evaluations using data from patients diagnosed with BP‐I showed that the previous popPK model accurately predicted plasma aripiprazole concentrations following administration of AOM during the double‐blind phase of the trial (ie, Phase D, the E‐R period; minimum of 2 doses of AOM) with better than required accuracy and bias. Collectively, these findings support that the inferences and the simulations based on the previous popPK model remained valid for the AOM 400 treatment arm of the trial in patients diagnosed with BP‐I.

Using the externally validated previous popPK model and the observed dosing histories in the BP‐I analysis dataset, model‐based exposure measures were calculated for use in the E‐R analysis. Per the final model, an E‐R relationship was established, such that each 1 ng/mL increase in aripiprazole was associated with a 0.34% decrease in the predicted hazard for recurrence of any mood episode. Importantly, the response of patients diagnosed with BP‐I to treatment with AOM exhibited a continuous relationship to aripiprazole exposure; this is in contrast to E‐R data in a schizophrenia population, in which a predicted aripiprazole C_min_ of 95 ng/mL was established as an important determinant of AOM efficacy.^2^ To allow for direct comparison and interpretation with the E‐R model in patients diagnosed with schizophrenia, a cut‐off of 95 ng/mL was tested as a new (categorical) model in the BP‐I population. Although results indicated that the 95 ng/mL cut‐off was a significant predictor of time to recurrence of any mood episode in patients diagnosed with BP‐I, the effect size was smaller compared with that in schizophrenia. That is, an aripiprazole C_tau_ of ≥95 ng/mL versus <95 ng/mL following the first dose in Phase D (a minimum of 2 doses of AOM) of the BP‐I trial was associated with a 36% or 1.55‐fold decrease in the predicted risk of recurrence of any mood episode; in comparison, a C_min_ of ≥95 ng/mL versus <95 ng/mL in a schizophrenia population was associated with a 4.41‐fold lower likelihood of relapse.^2^ An assessment of several other cut points in the BP‐I population (data not shown) demonstrated no meaningful difference compared with the 95 ng/mL cut‐point in terms of the predicted risk of recurrence of any mood episode, and none of the cut‐points described the data better than a continuous model.

It is not clear why the effect size of a 95 ng/mL cut‐point was lower in a BP‐I versus a schizophrenia population, but differences in disease symptomatology may play a role. Although psychotic symptoms can be present in both conditions, hallucinations and delusions are considered the hallmark symptoms of schizophrenia, while mood fluctuations are central to BP‐I.^8^ While there is robust evidence for the prophylactic efficacy of AOM 400 in preventing manic episodes in BP‐I,^7^ in the trial on which the current E‐R model is based, there were few depressive episodes during the study, with no difference observed between AOM and placebo for the recurrence of depressive episodes.^7^ While this could indicate a minimal effect of AOM on bipolar depression, the results could be explained by a floor effect driven by a patient population with minimal depressive symptoms at baseline.^7^ This is consistent with enrolment criteria that required patients to be experiencing a manic episode at enrolment, which is relevant since mood recurrence is more likely to be in the same polarity as the prior episode.^9^ Separate data from a multicenter, 1‐year, retrospective mirror‐image study showed a reduction in the number of depressive episodes, and the proportion of patients with depressive episodes decreased with AOM treatment.^10^ Taken together, further studies enrolling patients with an index manic episode or an index depressive episode may be considered to further elucidate the effect of aripiprazole on the prevention of depressive episodes in BP‐I compared with its mania‐preventing effects.

Data from the current E‐R modeling support current literature on the therapeutic reference range for aripiprazole (100–350 ng/mL),^11^ and provide a useful insight into the management of patients diagnosed with BP‐I who are receiving AOM and who might be at risk of subtherapeutic exposure to aripiprazole. The label for AOM does not include any requirement for therapeutic drug monitoring, and the results of the current analysis do not contradict this; that is, the lack of a simple 1:1 predictability of the E‐R model in terms of aripiprazole concentration versus effect means monitoring would be of no benefit in general. That said, plasma concentration monitoring on a case‐by‐case basis in patients with an inadequate clinical response to AOM remains relevant. In such a situation, if a patient was identified as having an aripiprazole plasma concentration of <95 ng/mL following 2 doses of AOM—placing them at an increased risk of a mood episode—reasons for this could be investigated (eg, missed/delayed dosing, ultrarapid CYP2D6 metabolism). Clinicians could then use this information to decide on a suitable management strategy (eg, adherence support interventions, oral aripiprazole supplementation, addition of a CYP2D6 inhibitor [if clinically indicated to target a specific symptom]). Having said this, data indicate that patients diagnosed with BP‐I who are treated with AOM will likely have sufficiently high plasma aripiprazole concentrations, along with maintained symptomatic stability.^5^ This is based on findings from an open‐label, 32‐week trial conducted in 266 patients with BP‐I or schizophrenia, which showed that most participants receiving AOM 400 or Ari 2MRTU 960 had plasma aripiprazole concentrations above 95 ng/mL, including for the entirety of the 2‐month dosing interval with Ari 2MRTU 960.^5^ A separate analysis conducted in the subpopulation of patients with BP‐I who participated in the study indicated that patients in both treatment groups remained clinically stable for the duration of treatment.^12^, ^13^

In the current analysis, time to recurrence of any mood episode was collected from patients following double‐blinded treatment with placebo or AOM. As such, an important characteristic was that measurable aripiprazole plasma concentrations were observed in the placebo treatment group during the beginning of the double‐blind period, related to the washout of the drug following administration of aripiprazole in the earlier phases of the trial. Exploratory analysis demonstrated that placebo‐treated patients had a higher probability (51.1%) of recurrence of any mood episode over time, as compared with patients administered AOM (26.5%). A similar trend was seen in the previously described E‐R analysis in patients diagnosed with schizophrenia, with a larger percentage of placebo versus AOM recipients experiencing a relapse (56.5% vs. 43.5%, respectively).^2^

Modeling efforts showed that a linear function of aripiprazole C_tau_ was a statistically significant predictor of the probability of time to recurrence of any mood episode, with none of the other tested covariates in addition to C_tau_ identified as statistically significant predictors. This indicates that plasma aripiprazole concentration is an important factor in predicting whether recurrence might occur in patients diagnosed with BP‐I, underscoring the importance of good adherence to AOM in maintaining consistent levels of plasma aripiprazole. To this end, clinicians should convey to patients the importance of treatment adherence, including how a reduction in plasma aripiprazole concentrations (eg, due to delayed or missed injections) could increase the risk of recurrence. Related to this, clinicians should explore any barriers to adherence (eg, difficulty attending appointments, a negative attitude toward treatment, health‐related challenges). This could include discussions regarding patients’ perceptions and attitudes toward how often treatment is administered, as a means of better tailoring treatment to improve medication adherence.^14^ Per the literature, patients may prefer LAIs that are dosed less often than once monthly (eg, because of less frequent injections, a feeling of receiving less medication, reduced burden of planning/administration).^14^, ^15^ Although these findings are specific to patients diagnosed with schizophrenia, they could feasibly apply to those with BP‐I, since both are long‐term mental illnesses with overlapping challenges regarding treatment adherence.^16^ In situations where patients indicate a preference for an LAI with an extended dosing interval, a switch from AOM to Ari 2MRTU 960 could be considered to reduce medication burden and enhance treatment adherence.^5^, ^6^, ^12^, ^13^ Ari 2MRTU 960 is indicated for the maintenance monotherapy treatment of adults diagnosed with BP‐I in the United States and some other countries, including Canada, and is administered via gluteal intramuscular injection once every 2 months.

## Conclusion

An E‐R analysis for AOM in patients diagnosed with BP‐I showed a continuous relationship between aripiprazole exposure and the risk of recurrence of any mood episode, with higher exposure associated with a lower recurrence risk. A separate categorical model using a plasma aripiprazole cut‐point of 95 ng/mL, established as an important efficacy threshold in schizophrenia, was also a significant predictor of time to mood episode recurrence in patients with BP‐I, although the effect size was smaller compared with that observed in schizophrenia. These findings confirm that plasma aripiprazole concentration is an important predictor of recurrence in patients diagnosed with BP‐I who are receiving AOM and emphasize the need to maintain therapeutic drug levels. Although therapeutic drug monitoring may be warranted in cases of inadequate response, it is not generally needed since most patients treated with AOM are expected to attain sufficiently high aripiprazole concentrations. The same is true for Ari 2MRTU 960, with most patients achieving therapeutic levels of aripiprazole across the full 2‐month dosing interval.

## Conflicts of Interest

Matthew Harlin and Karimah S. Bell Lynum are full‐time employees of Otsuka Pharmaceutical Development & Commercialization, Inc. At the time of the study, Xiaofeng Wang and Jessica Madera‐McDonough were full‐time employees of Otsuka Pharmaceutical Development & Commercialization, Inc. Pedro Such and Murat Yildirim are full‐time employees of H. Lundbeck A/S. At the time of the study, Frank Larsen was a full‐time employee of H. Lundbeck A/S. Julie Passarell is a full‐time employee of the Clinical Pharmacology and Pharmacometrics Business Unit of Simulations Plus, Inc., which was contracted to perform the analyses. At the time of the study, Luann Phillips was a part‐time employee of the Clinical Pharmacology and Pharmacometrics Business Unit of Simulations Plus, Inc.; she has since retired. Craig Chepke has served as an advisory board member for AbbVie, Acadia, Alkermes, Axsome, Biogen, Bristol Myers Squibb, Corium, Idorsia, Intra‐Cellular, Johnson & Johnson, Lundbeck, Moderna, Neurocrine, Noven, Otsuka, Sage, Sumitomo, and Teva (his spouse has served on advisory boards for Bristol Myers Squibb and Otsuka); has served as a consultant for AbbVie, Acadia, Alkermes, Axsome, Biogen, Boehringer Ingelheim, Corium, Intra‐Cellular, Johnson & Johnson, Lundbeck, MedinCell, Moderna, Neurocrine, Noven, Otsuka, Sage, Sumitomo, Supernus, and Teva; has received research/grant support from Acadia, Axsome, Harmony, Neurocrine, and Teva; has served on the speakers’ bureau for AbbVie, Acadia, Alkermes, Axsome, Bristol Myers Squibb, Corium, Intra‐Cellular, Johnson & Johnson, Lundbeck, Merck, Neurocrine, Noven, Otsuka, Sumitomo, and Teva; and has no stocks, ownership interests, or patents. Ric M. Procyshyn has served on the speakers’ bureau and advisory boards for AbbVie, Eisai, HLS Therapeutics, Janssen, Lundbeck, and Otsuka.

## Funding

The work described here was sponsored by Otsuka Pharmaceutical Development & Commercialization Inc. and H. Lundbeck A/S.

## Supporting information



Supporting Information

## Data Availability

To submit inquiries related to Otsuka clinical research or to request access to individual participant data (IPD) associated with any Otsuka clinical trial, please visit https://clinical‐trials.otsuka.com/. For all approved IPD access requests, Otsuka will share anonymized IPD on a remotely accessible data sharing platform.
