# Supplementary material for: Exposure–Response Analysis for Aripiprazole Once‐Monthly in Patients Diagnosed With Bipolar I Disorder
Source: Clin Pharmacol Drug Dev. 2025 Aug 22;14(11):869–78. doi: 10.1002/cpdd.1580 (PMC12583983; doi:10.1002/cpdd.1580)
Supplement: Supplementary file 1 — Supporting Information [file CPDD-14-869-s001.pdf]

Supplemental information

Exposure–Response Analysis For Aripiprazole Once-Monthly in Patients Diagnosed with Bipolar I Disorder

Xiaofeng Wang,[1] Luann Phillips,[2] Matthew Harlin,[1] Karimah S. Bell Lynum,[1] Frank Larsen,[3] Pedro Such, [3] Jessica Madera-McDonough, [1] Murat Yildirim,[3] Ric M. Procyshyn,[4] Craig Chepke,[5] and Julie Passarell[2]

[1]Otsuka Pharmaceutical Development & Commercialization, Inc., Princeton, NJ, USA; [2]Simulations Plus, Inc., Clinical Pharmacology and Pharmacometrics Business Unit, Buffalo, NY, USA; [3]H. Lundbeck A/S, Valby, Denmark; [4]Department of Psychiatry, University of British Columbia; British Columbia Mental Health and Substance Use Research Institute, Vancouver, BC, Canada; [5]Excel Psychiatric Associates, P.A., Huntersville, NC, USA

Corresponding author / address for reprints: Matthew Harlin, Otsuka Pharmaceutical Development & Commercialization, Inc., 2440 Research Blvd, Rockville, MD 20850, USA; work phone: +1 240-683-3055; e-mail: matthew.harlin@otsuka-us.com

Appendix S1. Trial Design, Eligibility Criteria, Study Sites, and Applicable Institutional Review Board(s)

Pharmacokinetic and efficacy data were derived from a Phase 3, 52-week, multicenter, randomized (1:1), placebo-controlled, double-blind clinical trial (NCT01567527) of aripiprazole once-monthly (AOM) as maintenance treatment in patients with bipolar I disorder (BP-I).[1] The trial had four phases: Phase A) if needed, conversion from other medications for BP-I to oral aripiprazole; Phase B) oral aripiprazole stabilization; Phase C) single-blind AOM stabilization; and Phase D) a double-blind withdrawal phase. Phase A (if warranted) was 4–6 weeks, Phase B was 2–8 weeks, Phase C was 12–28 weeks, and Phase D was 52 weeks. If a patient did not meet the goal of a study phase, they did not proceed to the next phase. The goal of Phase A was for all patients to achieve a monotherapy target starting dose of oral aripiprazole 15 mg/day. The goal of Phase B was to achieve pre-defined stability criteria during at least one bi-weekly visit during treatment with oral aripiprazole doses ranging from 15 to 30 mg/daily. Stability criteria were: 1) outpatient status; 2) Young Mania Rating Scale (YMRS) total score ≤12; 3) Montgomery–Åsberg Depression Rating Scale (MADRS) total score ≤12; and 4) no active suicidality, with active suicidality defined as a score of ≥4 on the MADRS item 10 or an answer of “yes” on questions 4 or 5 of the Columbia-Suicide Severity Rating Scale. During Phase C, patients were administered 10 or 15 mg of oral aripiprazole daily for the first 14 days (based on the final dose received in Phase B) and concomitantly administered AOM 400 mg (AOM 400) in the gluteal muscle. A single decrease to AOM 300 mg (AOM 300) was allowed for tolerability with a return to AOM 400, if needed. To move forward to Phase D, patients must have met the stability criteria for a minimum of 8 weeks without excursions (monitoring of stability began at Week 6 of Phase C). During the double-blind Phase D, patients were randomized (1:1) to continue the AOM treatment from Phase C or switch to placebo, stratified by region (North America, Europe, Japan, and other Asian countries). Another single-dose

modification to AOM 300 for tolerability or an increase back to AOM 400 was allowed, as needed. Oral aripiprazole was not administered during Phase D and patients were unaware of their transition from Phase C to Phase D. A diagram of the study design is provided in **Figure S1**.

The study population included male and female patients aged 18–65 years, inclusive. All patients had a diagnosis of BP-I according to Diagnostic and Statistical Manual of Mental Disorders, 4th Edition, Text Revision (DSM-IV-TR) criteria and confirmed by the Mini International Neuropsychiatric Interview. Patients must have experienced at least one previous manic or mixed episode with manic symptoms of sufficient severity to require hospitalization and/or treatment with a mood stabilizer and/or treatment with an antipsychotic agent in addition to a current manic episode. At entry in the trial, all patients had to be experiencing a manic episode (per DSM-IV criteria) with a YMRS total score ≥20. Both inpatients and outpatients were eligible for the trial. A list of investigational review boards for each of the 103 study sites is shown in **Table S1**.

Figure S1. Trial NCT01567527 Design Schematic.

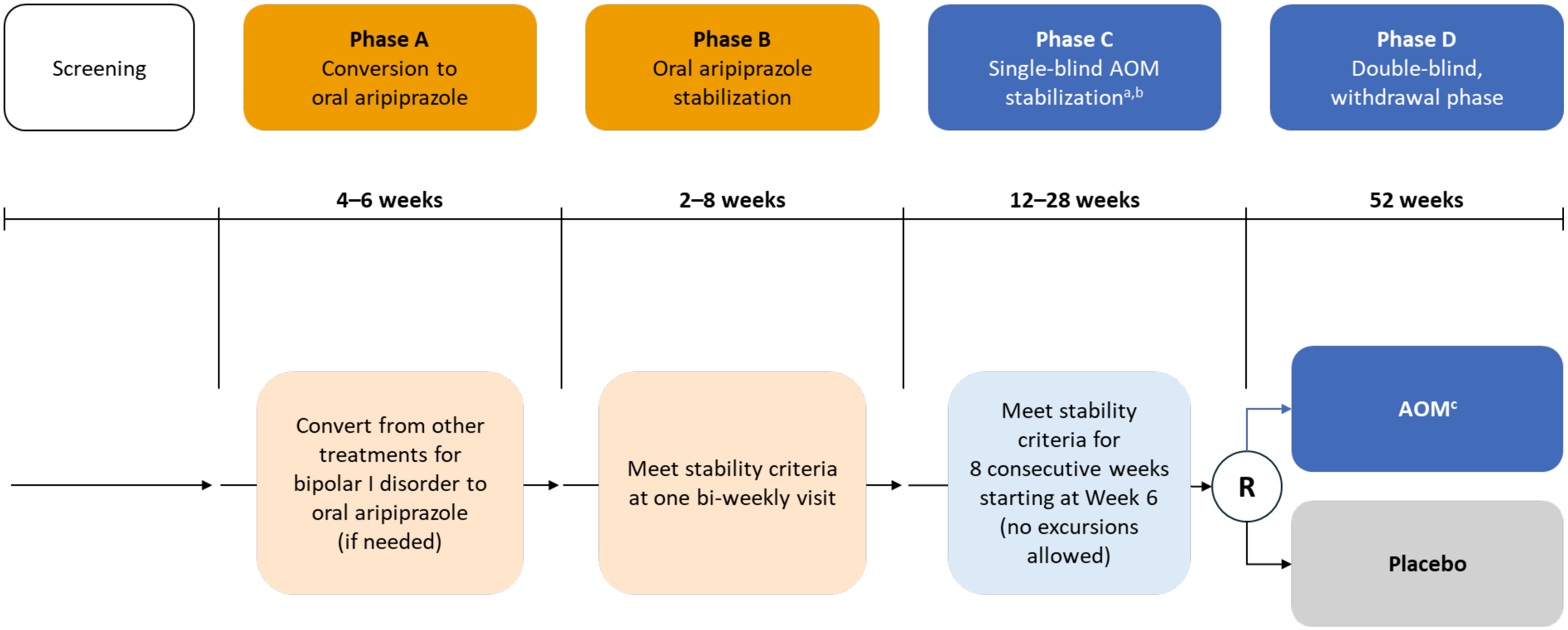

Note: 68 patients decreased from AOM 400 to AOM 300 (16.2% of patients) and 11 of those patients returned to AOM 400 during Phase C or Phase D of the trial.

[a]The starting dose was AOM 400 (a single decrease to AOM 300 was allowed for tolerability, with a return to AOM 400, if needed); [b]flexible dosing of oral aripiprazole was continued for the first 2 weeks of Phase C (10 mg/day if stable on 15–20 mg/day, 15 mg/day if stable on >20–30 mg/day); [c]the starting dose was AOM 400 or AOM 300, based on the final dose in Phase C (a single modification to the alternate dose and a return to the original dose was allowed, if needed).

AOM, aripiprazole once-monthly; AOM 300, aripiprazole once-monthly 300 mg; AOM 400, aripiprazole once-monthly 400 mg; R, randomization.

Table S1. List of institutional review boards according to study site number and location.

Site Number	Clinical Site	IRB	IRB Chair Name
001, 002, 003, 004, 005, 006, 007, 008, 009, 010, 011, 012, 013, 015, 016, 017, 019, 020, 021, 022, 023, 025, 027, 028, 029, 030, 033, 034, 035, 036, 037, 038, 039, 042, 043, 045, 046, 048, 049, 052, 053, 054, 055, 056, 057, 058, 059, 060, 061, 064, 065, 066, 067, 068, 069	USA sites, Central IRB	Schulman Associates Institutional Review Board, 4445 Lake Forest Drive, Suite 300, Cincinnati, Ohio 45242, USA	Sharon Lynn Nelson, MSN, RN, CNS
014	USA, Local IRB	UC Irvine: Office of Research Institutional Review Board, 5171 California, Suite 150, Irvine, California 92697, USA	Kenneth G. Linden, M.D., Ph.D.
024	USA, Local IRB	University at Buffalo: Health Sciences Institutional Review Board, 875 Ellicott Street, Buffalo, New York 14203, USA	Ron Moscati
040	USA, Local IRB	Western Institutional Review Board, 3535 7th Avenue SW, Olympia, Washington 98502, USA	Bert Wilkins, J.D., MHA

047	USA, Local IRB	Louisiana State University Health Sciences Institutional Review Board, 433 Bolivar Street Suite 206, New Orleans, Louisiana 70112, USA	Kenneth E. Kratz, Ph.D.
051	USA, Local IRB	Western Institutional Review Board, 3535 7th Avenue SW, Olympia, Washington 98502, USA	Bert Wilkins, J.D., MHA
080, 081	Canada, Central IRB	Schulman Associates Institutional Review Board, 4445 Lake Forest Drive, Suite 300, Cincinnati, Ohio 45242, USA	Sharon Lynn Nelson
100, 102, 103, 104	Poland, Central IRB	Komisja Bioetyczna przy Bydgoskiej lzbie Lekarskiej ul. Powstadcow Warszawy 1 1 85-681 Bydgoszcz, Poland	Chair Name not provided
150, 151, 152, 153, 154, 155	Romania, Central IRB	Comisia Nationala de Etica pentru Studiul Clinic al Medicamentului Str. Av. Sanatescu nr.48 Sector I Bucuresti, Romania	Chair Name not provided
200	Taiwan, Local IRB	Taipei City Hospital Institutional Review Board No. 145, Zhengzhou Road, Taipei City, 103, Taiwan	Kuang-Shen Li
201, 202	Taiwan, Local IRB	Chang Gung Medical Foundation Institutional Review Board, No. 199, Tunhua North Road, Taipei City, 105, Taiwan	Tsang Tang
250	South Korea, Local IRB	Institutional Review Board of The Catholic University of Korea 10, 63-ro, Yeongdeungpo-gu, 150-713, Republic of Korea	Ki-Sung Ryu

251	South Korea, Local IRB	Institutional Review Board of Eulji General Hospital 68, Hangeulbiseok-ro, Nowongu, Seoul, 139-711, Republic of Korea	Kwon, O Hyun
252	South Korea, Local IRB	Institutional Review Board of Jeju National University Hospital Aran 13gil 15, Jeju-si, Jeju Special Self-Governing Province, 690-767, Republic of Korea	Lee, Chang Sub
253	South Korea, Local IRB	Institutional Review Board of Chungnam National University Hospital 282, Munhwa-ro, Jung-gu, Daejeon, 30”1-721, Republic of Korea	Suhm Kwang-Sun
254	South Korea, Local IRB	Institutional Review Board of Korea University Anam Hospital 73 Inchon-ro, Seongbuk-Gu, Seoul, 136-705, Republic of Korea	Jeen, Yoon Tae
255	South Korea, Local IRB	Institutional Review Board of Dongguk University Ilsan Hospital 27 Dongguk-rom, Ilsandong-gu, Goyang-si, 410-773, Republic of Korea	Kim Eung-Jung
256	South Korea, Local IRB	Institutional Review Board of Hallym University Sacred Heart Hospital 22, Gwanpyeong-ro 170beon-gil, Dongan-gu, Anyang-si, Gyeonggi-do, 431-070, Republic of Korea	Kim Kwang Nam

300	Japan, Local IRB	Yuge Hospital Institutional Review Board, 679-2 Yuge, Tatsuda-machi, Kita-ku, Kumamoto prefecture 861-8002, Japan	Midori Suematsu
301	Japan, Local IRB	Arakaki Hospital Institutional Review Board, 4-10-3 Ageda, Okinawa, 904-0012, Japan	Norifumi Kunimoto
302	Japan, Local IRB	South Toyama Nakagawa Institutional Review Board, 146 Omachi, Toyama city, Toyama prefecture, 939-8073, Japan	Kazuhiko Katsukawa
303	Japan, Local IRB	NHO Tottori Medical Center Institutional Review Board, 876 Mitsu, NHO Tottori Medical Center, Tottori city, Tottori prefecture, 689-0203, Japan	Tsuruhei Sukegawa
304	Japan, Local IRB	Goryokai Medical Corporation Institutional Review Board, 6-2-3 9-jo, Shinoro, Kita-ku, Sapporo city 002-8029, Hokkaido, Japan	Umeko Sakaoka
305	Japan, Local IRB	Asai Clinic Institutional Review Board, 1-14 Katabira-cho, Hodogaya-ku, Yokohama 240-0013, Kanagawa, Japan	Junichi Kaburaki
306	Japan, Local IRB	Asakayama General Hospital Institutional Review Board, 3-3-16 Imaike-cho, Sakai-ku, Sakai city 590-0018, Osaka, Japan	Ryo Takahashi
308	Japan, Local IRB	Nara Medical University Institutional Review Board, 840 Shijo-cho, Kashihara 634-8522, Nara, Japan	Masatoshi Hasegawa

309	Japan, Local IRB	Hoshi General Hospital Institutional Review Board, 159-1 Mukaikawara-machi, Koriyama 963-8501, Fukushima, Japan	Kaori Hirai
311	Japan, Local IRB	Shinagawa East One Medical Clinic Institutional Review Board, 2-16-1, Konan Minato-ku, Tokyo 108-0075, Japan	Hideaki Sakai
312, 313	Japan, Local IRB	Seiwakai Medical Corporation Association Yutaka Clinic Institutional Review Board, 3-14-20 Sagamiono, Minami-ku, Sagamihara-shi 252-0303, Kanagawa, Japan	Soichiro Watanabe
314	Japan, Local IRB	Tokyo Women's Medical University Institutional Review Board, 8-1 Kawada-cho, Shinjyuku-ku 162-8666, Tokyo, Japan	Jun Ishigooka
315	Japan, Local IRB	Medical Corporation Kyouwakai, Hannan Hospital Institutional Review Board, 277, Handa Minamino-cho, Naka-ku, Sakai 599-8263, Osaka, Japan	Tetsuya Inoue
317	Japan, Local IRB	Arakaki Hospital Institutional Review Board, 4-10-3 Ageda, Okinawa, 904-0012, Japan	Norifumi Kunimoto
318	Japan, Local IRB	Medical Corporation Houmankai Umezu Clinic Institutional Review Board, 2-6-12 Harada, Chikushino 818-0024, Fukuoka, Japan	Tetsuji Inou

319	Japan, Local IRB	Takeda General Hospital Institutional Review Board, 3-27 Yamagamachi, Aizu Wakamatsu 965-8585, Fukushima, Japan	Kouichi Osonoe
320	Japan, Local IRB	Fukuoka University Hospital Institutional Review Board, 7-45-1 Nanakuma, Jyonan-ku, Fukuoka-shi 814-0180, Fukuoka, Japan	Toshihiko Yanase
321	Japan, Local IRB	Iwate Medical University Institutional Review Board, 19-1m Uchimaru, Morioka-shi 020-8505, Iwate, Japan	Toru Sugiyama
322	Japan, Local IRB	National Hospital Organization Hizen Psychiatric Center Institutional Review Board, 160, Mitsu, Yoshinogari-cho, Kanzaki-gun 842-0192, Saga, Japan	Kijiro Hashimoto
323	Japan, Local IRB	Nagasaki Medical Center of Psychiatry Institutional Review Board, 1575-2 Seibu Machi, Omura 856-0847, Nagasaki, Japan	Kyoko Kubo

IRB, institutional review board.

Appendix S2. Previously Developed Population Pharmacokinetic Model

The previously developed population pharmacokinetic (popPK) model was a 3-compartment model with first-order elimination and sigmoid-absorption (zero-order input to the depot compartment and first-order absorption to the central) for oral aripiprazole and first-order absorption for AOM. The AOM absorption rate constant decreased with increasing body mass index and was approximately 35% higher for males. The clearance was slower for patients with cytochrome P450 (CYP)2D6 poor metabolizer status and for patients with co-administration of strong CYP2D6 or CYP3A4 inhibitors. The AOM formulation was estimated to be 1.48 times more bioavailable than the oral formulation. A diagram of the model is shown in **Figure S2**, while parameter estimates and standard errors are shown in **Table S2**. Further details are provided in the publication detailing the previous popPK model.[2]

Figure S2. Previous Population Pharmacokinetic Model Diagram.

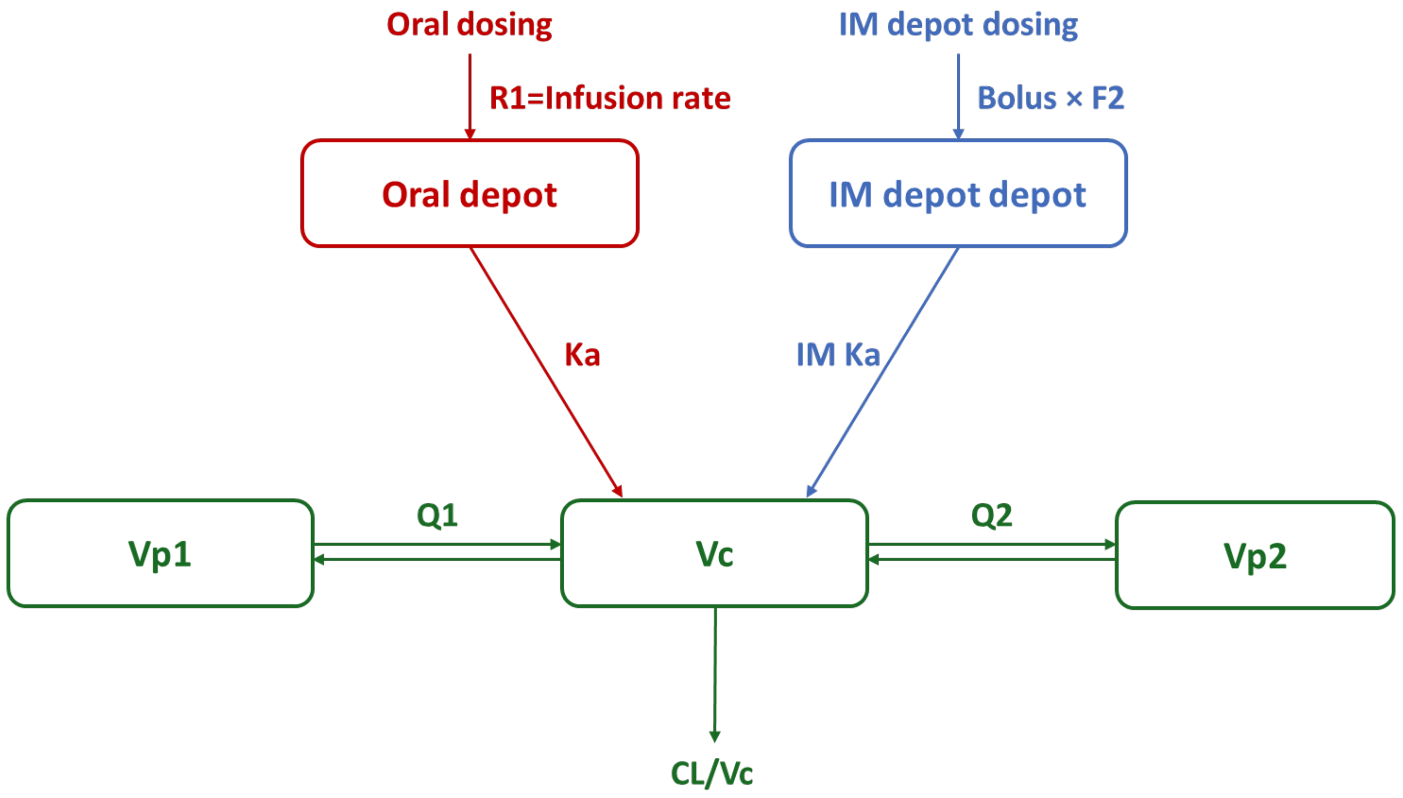

Bolus, bolus injection of IM depot immediately into the IM depot compartment (typical first-order absorption of IM depot dosing); CL, apparent clearance; F2, relative bioavailability of IM depot as compared to oral aripiprazole; IM, intramuscular; IM Ka, first-order absorption rate constant for IM administration (function of body mass index and sex); Ka, oral absorption rate constant; Q1, intercompartmental clearance between central and first peripheral; Q2, intercompartmental clearance between central and second peripheral; R1, infusion rate of oral dosing to the oral depot compartment (allows sigmoid absorption); Vc, apparent central volume of distribution; Vp1, apparent volume of distribution for first peripheral compartment; Vp2, apparent volume of distribution for second peripheral compartment.

Table S2. Parameter Estimates and Standard Errors for the Previously Developed Population Pharmacokinetic Model for Oral Aripiprazole and Aripiprazole Once-Monthly.

Parameter	Final parameter estimate		Magnitude of interindividual variability (%CV)[a]	
	Population Mean	%SEM	Final Estimate	%SEM
**K_a: Oral first-order absorption rate (1/h)**	0.540	FIXED	65.88	Fixed
**CL: Clearance for EM (L/h)[b]**	3.71	4.0	38.34	6.9
**CL: Clearance for PM (L/h)**	1.88	6.9		
**CL: Proportional change in CL for CYP2D6 inhibitor**	-0.511	FIXED		
**CL: Proportional change in CL for CYP3A4 inhibitor**	-0.237	FIXED		
**V_c: Central volume (L)**	93.4	8.8	124.50	15.2
**Q1: Inter-cmt CL (L/h)**	0.591	FIXED	NE	NA
**Vp1: Peripheral volume (L)**	118	FIXED	NE	NA
**Q2: Second inter-cmt CL (L/h)**	28.8	FIXED	NE	NA
**Vp2: Second peripheral volume (L)**	134	FIXED	NE	NA
**R1: Rate of dose into depot (mg/h)**	9.33	FIXED	NE	NA
**IM K_a: IM depot first-order absorption rate (1/h)**	0.000904	5.3	55.59	8.2
**F2: Relative bioavailability for IM depot[b]**	1.48	4.9	NE	NA
**IM K_a: Power for (BMI/28)**	-0.975	11.5	NE	NA
**IM K_a: Proportional shift for males**	0.346	28.9	NE	NA
**Phase 1 RV (%CV)**	24.23	8.4	NA	NA
**Phase 3 RV (%CV)**	28.11	4.7	NA	NA
Minimum value of the objective function = 48892.907				

Note: Model development analysis dataset contained Phase 1 and Phase 3 trials with oral and IM depot dosing.

[a]The shrinkage for the interindividual variability of Ka, CL, Vc, and IM Ka was 73.9%, 7.3%, 52.2%, and 19.5%, respectively; [b]the estimate of the clearance for extensive metabolizers was correlated with the estimate of the relative bioavailability for IM depot (r = 0.901).

$$IMKa = \left[0.000904 \times \left(\frac{BMI}{28}\right)^{-0.975}\right] \times (1 + 0.346 \times Males)$$

$$CL = (3.71 \times EM + 1.88 \times PM) \times (1 - 0.511 \times CYP2D6\ Inhibitor) \times (1 - 0.237 \times CYP3A4\ Inhibitor)$$

Where: Each discrete population descriptor is 1 for patients with the matching descriptor value and is 0 otherwise.

cmt, compartmental; %CV, percent coefficient of variation; F2, relative bioavailability; IM, intramuscular; NA, not applicable; NE, not estimated; Q1, intercompartmental clearance between central and first peripheral; Q2, intercompartmental clearance between central and second peripheral; R1, infusion rate of the oral dose into the Depot compartment; RV, residual variability; %SEM, percent standard error of the mean; Vp1, apparent volume of distribution for first peripheral compartment; Vp2, apparent volume of distribution for second peripheral compartment.

Table reproduced from Wang X, Raoufinia A, Bihorel S, et al. Population pharmacokinetic modeling and exposure–response analysis for aripiprazole once monthly in subjects with schizophrenia. © 2022 The Authors. *Clinical Pharmacology in Drug Development* published by Wiley Periodicals LLC on behalf of American College of Clinical Pharmacology. The article is licensed under the Creative Commons Attribution License (CC BY 4.0), which permits use, distribution, and reproduction in any medium, provided the original work is properly cited. To view a copy of this license, visit https://creativecommons.org/licenses/by/4.0/. Original article available at: https://accp1.onlinelibrary.wiley.com/doi/10.1002/cpdd.1022.

Appendix S3. Validation of the Previously Developed Population Pharmacokinetic Model

Methodology

1: Applying the population pharmacokinetic model to the bipolar I disorder trial data

A sample to determine the CYP2D6 metabolizer status was not collected from all patients in the BP-I trial. Therefore, during the first step of applying the previously developed popPK model (all parameters fixed to the previous estimates), an additional mixture model subroutine ($MIX) was used to allow the model to estimate whether patients with an unknown CYP2D6 metabolizer status were more likely to have originated from a patient with poor metabolizer or ultra, extensive, and intermediate metabolizer status. This assignment was then used to apply the popPK model a final time to obtain population-predicted plasma concentrations (PRED; does not include inter-individual variability [IIV]) and individual-predicted plasma concentrations (IPRED; includes IIV) for patients diagnosed with BP-I.

2: Quantitative predictive performance validation

To provide a quantitative assessment of the model fit, individual percent prediction errors (%IPEs) and population percent prediction errors (%PEs) were calculated to measure the bias of the predicted plasma concentrations. The absolute values of the %PEs and %IPEs (|%PEs| and |%IPEs|) were calculated to measure the accuracy in the predictions. Prior to the analysis, it was determined that a median %PE within ± 10% and a median |%PE| <40% would indicate that the previous popPK model was adequate to describe the BP-I data.

$$\%IPE = 100 \times (DV - IPRED)/IPRED$$

$$\%PE = 100 \times (DV - PRED)/PRED$$

3: Visual predictive check validation

Assuming that uncertainty in the final popPK model parameters was small relative to other sources of variability, the adequacy of the previous popPK model to describe the data from the current trial was also evaluated using a prediction-corrected visual predictive check method.[3] The previous popPK model was used to simulate 1000 replicates of the current BP-I analysis dataset. The 5th, 50th (median), and 95th percentiles from the simulated and observed data were calculated for comparison. These percentiles of the plasma concentration data versus time since first dose — with the observed data and percentiles of the observed data — were then overlaid to visually assess concordance between the model-based simulated data and the observed data. If the visual concordance of the simulated and observed data was adequate and the quantitative predictive performance validation was sufficient, the previous popPK model would be appropriate to predict the pharmacokinetic exposures for the exposure–response (E-R) model.

Results

Baseline demographic data for the popPK population are provided in **Table S3**.

As shown in **Table S4**, the median population percent prediction error (%PE) of -10.09% was slightly below the predefined criteria for bias (± 10%). However, the median absolute prediction error (|%PE|) of 34.55% was within the predefined criteria for accuracy (40%). As a further assessment, the %PE and |%PE| were calculated for samples collected during Phase D of the trial only. The median %PE was -2.01% and the median |%PE| was 32.01%, which were within the predefined criteria for accuracy and precision. In addition, prediction-corrected visual predictive checks (pcVPCs) (**Figure S3** and **Figure S4**) showed that the simulated and observed percentiles exhibited good correspondence after the second dose of AOM (post 1400 hours). There was some overprediction bias during the concurrent dosing of oral aripiprazole and AOM (<1400 hours; during Phase C), which was most likely a result of the assumption of steady-state conditions at time since first dose of 0 hours. Across the

full range of time, 8.86% and 3.93% of the observed data were below the simulated 5th percentile and above the 95th percentile, respectively. For time >1400 hours, 5.19% and 4.50% of the observed data were below the simulated 5th percentile and above the 95th percentile, respectively. The results of the pcVPC indicated that the previous popPK model predicted the plasma concentrations and the variability of the concentrations well after the first 1400 hours (approximately 58 days) of dosing (two AOM doses; end of Phase C and Phase D). Because the primary focus for this analysis was the prediction of exposures for the E-R analysis (Phase D of the trial), the median %PE of the full analysis dataset was near the accepted limit, and the pcVPC exhibited a good correspondence after two AOM doses, the model predictions were considered acceptable for accuracy and precision.

Table S3. Baseline Demographic Characteristics of Patients Included in the Population Pharmacokinetic Model (N=420).

**Patient characteristic**	**N=420**
**Age, years**	40.0 (11.3)
**Weight, kg**	87.17 (22.5)
**Body mass index, kg/m^2**	30.5 (7.2)
**Sex, n (%)**	
**Male**	167 (39.8)
**Female**	253 (60.2)
**Race, n (%)**	
**White**	232 (55.2)
**Black/African American**	117 (27.9)
**Asian**	55 (13.1)
**American Indian or Alaska native**	3 (0.7)
**Other**	12 (2.9)
**Unknown**	1 (0.2)
**Ethnicity, n (%)**	
**Hispanic or Latino**	30 (7.1)
**Not Hispanic or Latino**	390 (92.9)
**CYP2D6 metabolizer status, n (%)**	
**Intermediate, extensive, ultra rapid**	381 (90.7)
**Poor**	12 (2.9)
**Inconclusive or missing**	27 (6.4)

Data are shown as mean (standard deviation) unless otherwise stated.

CYP, cytochrome P450.

Table S4. Summary Statistics of Population and Individual Prediction Errors.

Variable	Number of Samples	Minimum	5th Percentile	25th Percentile	Median	75th Percentile	95th Percentile	Maximum	Mean	SD
**Full Population PK Analysis Dataset Meeting Steady-State Model Requirements**										
**Population Prediction Error (%)**	1907	-95.6	-70.8	-40.6	-10.09	25.9	109.4	370.1	-0.06	57.38
**Absolute Population Prediction Error (%)**	1907	0.0	2.8	15.8	34.55	58.9	109.4	370.1	42.71	38.31
**Individual Prediction Error (%)**	1907	-91.8	-45.4	-11.3	3.55	16.5	35.2	83.3	1.05	24.37
**Absolute Individual Prediction Error (%)**	1907	0.0	1.5	7.0	14.65	26.0	50.9	91.8	18.55	15.84
**Samples Collected During Phase D of the Trial Only**										
**Population Prediction Error (%)**	588	-80.7	-62.3	-28.2	-2.01	37.5	114.9	270.5	8.60	54.23
**Absolute Population Prediction Error (%)**	588	0.1	2.4	16.0	32.01	55.8	114.9	270.5	41.14	36.33
**Individual Prediction Error (%)**	588	-83.6	-25.6	-3.1	8.49	20.3	37.9	83.3	8.03	19.97
**Absolute Individual Prediction Error (%)**	588	0.0	1.1	6.7	13.54	23.6	41.6	83.6	16.68	13.60

PK, pharmacokinetic; SD, standard deviation.

Figure S3. Prediction-corrected visual predictive check of the previously developed population pharmacokinetic model applied to the current analysis dataset meeting steady-state model requirements. Note: medians and percentiles are plotted at the median time since first dose of the data observed within each time since first dose interval. CI, confidence interval.

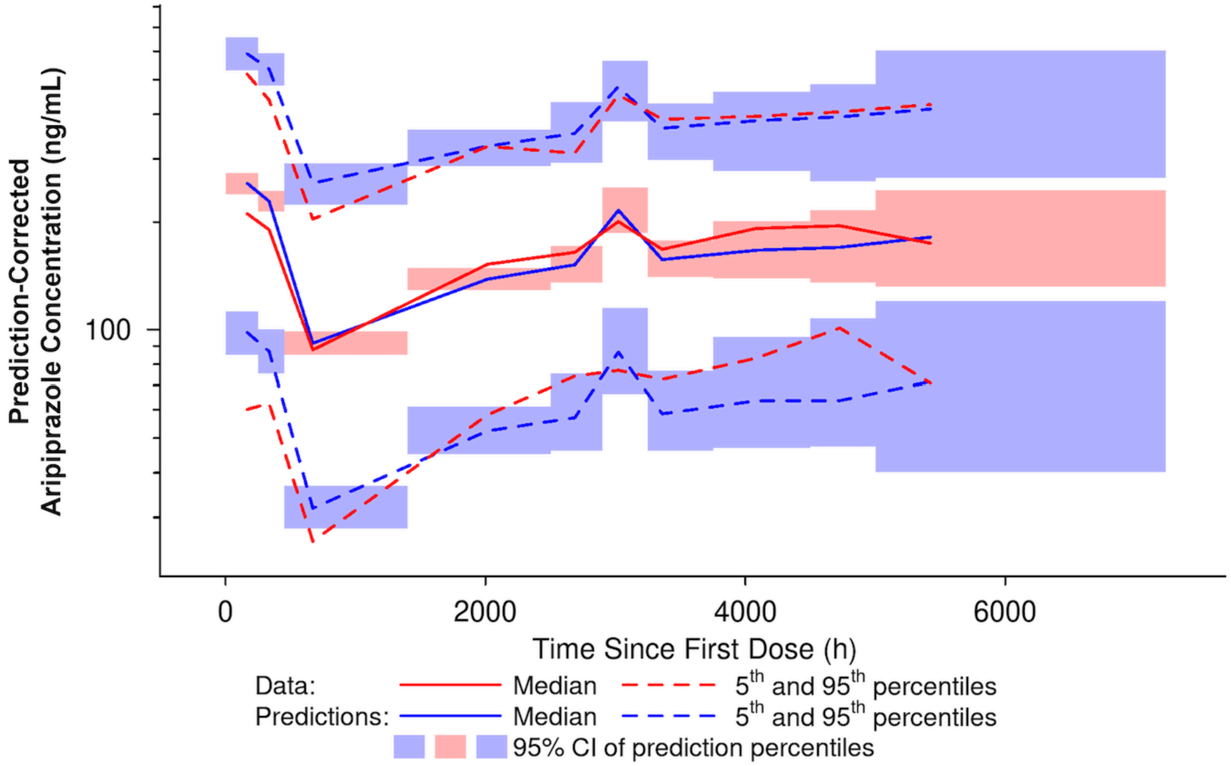

Figure S4. Prediction-corrected visual predictive check of the previously developed population pharmacokinetic model applied to the current analysis dataset meeting steady-state model requirements, with the observed data overlaid. Note: medians and percentiles are plotted at the median time since first dose of the data observed within each time since first dose interval. CI, confidence interval.

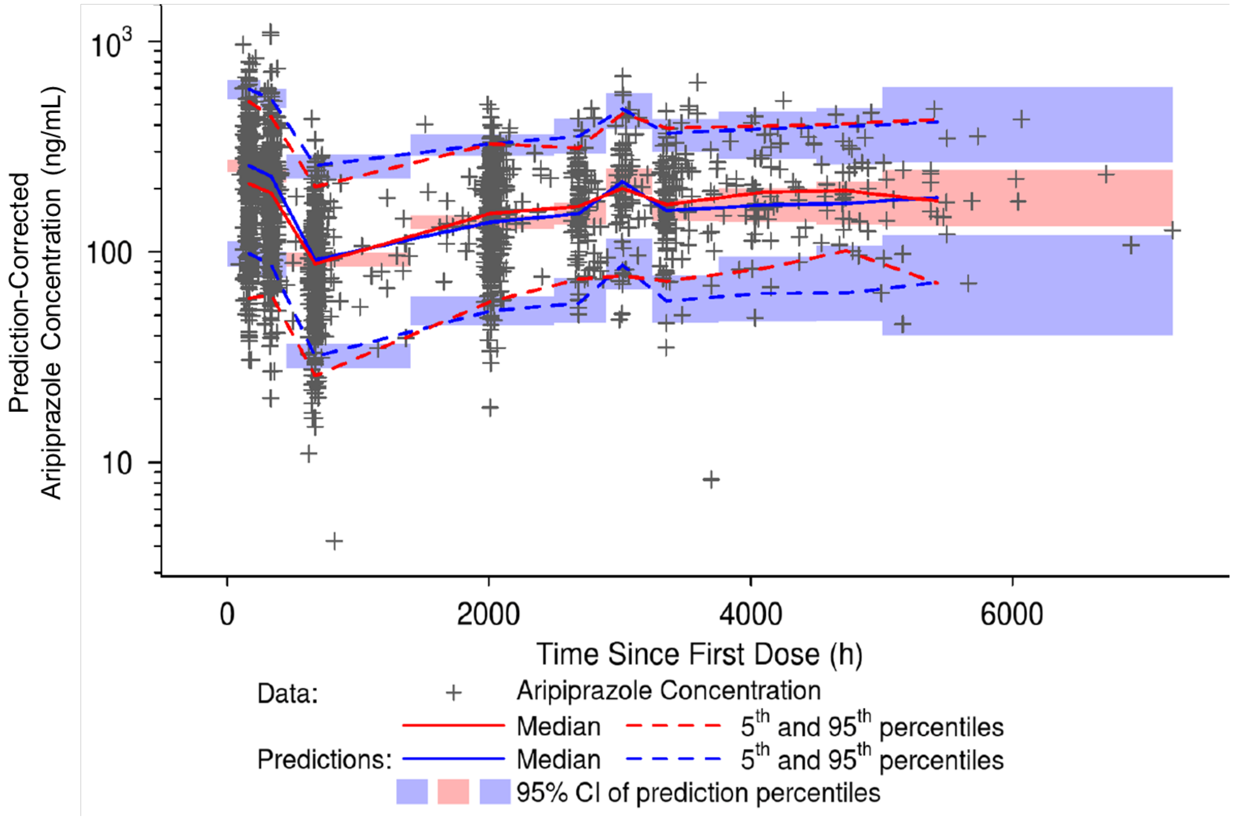

Appendix S4. Development of the Exposure–Response Model

1: Exploratory data analysis

A summary of demographic characteristics for the E-R efficacy analysis population, stratified by treatment, is provided in **Table S5**.

Exploratory data analyses and data visualization techniques were used to understand the informational content of the dataset with respect to the anticipated model and for verification of various model assumptions. In addition, the exploratory analyses allowed for the potential identification of trends in the data, to be used to determine the appropriate functional forms used to evaluate the relationship of the probability of survival (i.e., no mood episode) over time to aripiprazole plasma concentration 672 hours after the first dose of AOM (C_{tau}).

2: Base structural model development

A semi-parametric Cox proportional hazards model was used to explain the effect of drug exposure (i.e., C_{tau}) on the log of the survival function. The assumption for the Cox proportional hazards model using the correlation between the time to recurrence of any mood episode and the Schoenfeld residuals was tested and met. For each model, R computed minus twice the log likelihood of the data. In the case of hierarchical models, the change in minus twice the log likelihood produced by the inclusion of a parameter is asymptotically $\mathcal{X}^2$-distributed, with the number of degrees of freedom (df) equal to the number of parameters added to or deleted from the model. A decrease in minus twice the log likelihood of at least 3.84 ($\alpha = 0.05$, 1 df) was used to define statistical significance for the addition of a single parameter. The criteria for goodness of fit and model selection included (but were not limited to) examination of the following: agreement in Kaplan–Meier (KM) plots of the observed and predicted probability of survival overall, and stratified by categorical predictor variables or for various groupings of continuous predictor variables;

improvement in the precision of parameter estimates in the survival model, as measured by the relative standard error (RSE) expressed as a percent (%RSE = standard error/parameter estimate × 100); and comparison of minus twice the log likelihood values for hierarchical models.

3: Covariate analysis

Following the development of the base structural model incorporating C_{tau}, the influence of covariates on the time to event was evaluated. To avoid potential multicollinearity or confounding of effects in covariate submodels, the correlation between covariates was examined prior to covariate analysis (none were found).

A full model was evaluated, incorporating aripiprazole C_{tau} and all covariate effects additively into the model. The resultant model was considered the full survival model. Using the full model, univariate stepwise backward elimination was performed by removing each covariate one at a time. A covariate was considered significant if it resulted in a change in the value of the objective function of ≥6.84 ($P < 0.01$, 1 df for $\mathcal{X}^2$-distribution) when removed from the model. The most non-significant covariate (i.e., the one with the highest $P > 0.01$) was removed from the model first, with the reduced model then serving as the new base multivariable model. The backward elimination procedure was repeated until all remaining covariates were significant at $\alpha = 0.01$.

4: Exposure–response model evaluation

Plots of the model-predicted probability of survival over time, overlaid with the observed KM estimates, were examined to evaluate the quality of the final model. In addition, the adequacy of the final E-R model was evaluated using a simulation-based visual predictive check method. Using the E–R analysis population, patients were randomly re-sampled with replacement to create 500 replicates of the analysis dataset with R software. Using the final E-R model for time to event with the replicated data, the predicted survival at each time

interval was calculated given the covariates and predicted C_{tau}. A uniform random variable was used to determine if a patient had an event during that time interval by comparing the random variable to the predicted survival at that time. If the random variable was larger than the predicted survival, the patient was flagged as having an event at that time. The 5th, 50th (median), and 95th percentiles of the distributions of the simulated percent of patients without the event at each time for each group of exposure were calculated and were compared with the original observed data to visually assess concordance of the model-based simulated and observed data.

5: Exposure–response final model

The final E-R model for time to recurrence of any mood episode was a semi-parametric Cox proportional hazards model including the effect of first-dose Phase D aripiprazole C_{tau} as shown in the following equation:

$$S(t) = S_0(t)^{[exp(-0.00346 \times Ctau_i)]}$$

Where: $S_0(t)$ is the baseline survival function of recurrence of any mood episode at time t; and $Ctau_i$ is the aripiprazole C_{tau} following the first dose of AOM in Phase D in the *i*th patient.

Table S5. Baseline demographic characteristics of patients included in the exposure–response analysis (N=265)

Patient characteristic	N=265
**Age, years**	40.1 (11.0)
**Body mass index, kg/m^2**	30.2 (7.2)
**MADRS total score**	2.7 (3.4)
**YMRS total score**	2.8 (3.3)
**Sex, n (%)**	
**Male**	113 (42.6)
**Female**	152 (57.4)
**Race, n (%)**	
**White**	144 (54.3)
**Black/African American**	74 (27.9)
**Asian**	37 (14.0)
**American Indian or Alaska native**	2 (0.8)
**Other**	8 (3.0)
**Number of psychiatric co-medications, n (%)**	
**0**	122 (46.0)
**1**	76 (28.7)
**2**	49 (18.5)
**3**	13 (4.9)
**4**	3 (1.1)
**5**	1 (0.4)
**6**	1 (0.4)

Data are shown as mean (standard deviation) unless otherwise stated.

MARDS, Montgomery–Åsberg Depression Rating Scale; YMRS, Young Mania Rating Scale.

References

1. Calabrese JR, Sanchez R, Jin N, et al. Efficacy and safety of aripiprazole once-monthly in the maintenance treatment of bipolar I disorder: a double-blind, placebo-controlled, 52-week randomized withdrawal study. *J Clin Psychiatry*. 2017;78(3):324–331.

2. Wang X, Raoufinia A, Bihorel S, et al. Population pharmacokinetic modeling and exposure–response analysis for aripiprazole once monthly in subjects with schizophrenia. *Clin Pharmacol Drug Dev*. 2022;11(2):150–164.

3. Nguyen TH, Mouksassi MS, Holford N, et al. Model evaluation of continuous data pharmacometric models: metrics and graphics. *CPT Pharmacometrics Syst Pharmacol*. 2017;6(2):87–109.
